# Responding to aid volatility: government spending on district health care in Zambia 2006–2017

**DOI:** 10.1080/16549716.2020.1724672

**Published:** 2020-02-19

**Authors:** Amy Jackson, Birger Forsberg, Collins Chansa, Jesper Sundewall

**Affiliations:** a Department of Global Public Health, Karolinska Institutet, Stockholm, Sweden; b Institute of Global Health, Heidelberg University, Heidelberg, Germany; c HEARD, University of KwaZulu-Natal, Durban, South Africa

**Keywords:** Primary care, health financing, official development assistance, aid coordination, fungibility, decentralisation, corruption, aid effectiveness

## Abstract

**Background**: A corruption event in 2009 led to changes in how donors supported the Zambian health system. Donor funding was withdrawn from the district basket mechanism, originally designed to pool donor and government financing for primary care. The withdrawal of these funds from the pooled financing mechanism raised questions from Government and donors regarding the impact on primary care financing during this period of aid volatility.

**Objectives**: To examine the budgets and actual expenditure allocated from central Government to the district level, for health, in Zambia from 2006 to 2017 and determine trends in funding for primary care.

**Methods**: Financial data were extracted from Government documents and adjusted for inflation. Budget and expenditure for the district level over the period 2006 to 2017 were disaggregated by programmatic area for analysis.

**Results**: Despite the withdrawal of donor funding from the district basket after 2009, funding for primary care allocated to the district level more than doubled from 2006 to 2017. However, human resources accounted for this increase. The operational grant, on the other hand, declined.

**Conclusion**: The increase in the budget allocated to primary care could be an example of ‘reverse fungibility’, whereby Government accounted for the gap left by donors. However, the decline in the operational grant demonstrates that this period of aid volatility continued to have an impact on how primary care was planned and financed, with less flexible budget lines most affected during this period. Going forward, Government and donors must consider how funding is allocated to ensure that primary care is resilient to aid volatility; and that the principles of aid effectiveness are prioritised to continue to provide primary health care and progress towards achieving health for all.

## Background

Renewed efforts towards achieving universal health coverage by 2030 are only possible with continued attention and prioritisation of primary health care. The 1978 declaration of Alma-Ata highlights this, stating that primary health care is the key to achieving ‘health for all’ [1, p. 2]. Primary care is defined as the provision of first contact, person focussed care, that is able to deal with most health needs [2, p. 458]. Despite the recognised importance of primary care, many low and middle-income countries have failed to provide a quality primary care package of essential services to its citizens [3]. Primary care expenditure has not reflected the status given to it in the global community, with funding described as ‘insufficient and inconsistent’ [4, p. 322].

Primary care has been noted to benefit particularly from the aid effectiveness agenda, with the OECD stating that increased aid coordination is correlated with the increased coverage and use of primary care. Declarations and commitments, including the Paris Declaration of 2005 and the International Health Partnership Plus (IHP+), have enshrined the importance of aid effectiveness, with particular emphasis on the principles of government ownership, alignment and harmonisation [5]. Attempts to implement these principles have included Sector Wide Approaches (SWAps) and specific finance mechanisms, including general budget support and sector budget support, and, at least on paper, have been enthusiastically adopted by both donor and recipient governments [6,7].

In Zambia, donors, known as ‘Cooperating Partners’ (CPs), up until 2009, channelled funds into a basket mechanism. The term ‘basket funding’ in Zambia refers to the co-financing of district health services by a number of donors and government using a single set of procedures [8]. Channelling domestic and international funds directly to districts for primary care, through the basket, ensured that CPs’ support was aligned with the Government’s priority of providing ‘equity of access to cost-effective, quality healthcare services as close to the family as possible,’ or primary care. The adoption of on-budget support enabled government to exercise strong ownership of the aid in the health sector and provides an example of how the principles of the aid effectiveness agenda can be put in to practice [9].

However, challenges in implementation have been notable. Zambia provides an example whereby donors have withdrawn direct financial aid to Government in response to government corruption. Following a corruption event in 2009, involving Ministry of Health officials, CPs froze funding to the basket mechanism [10]. As a result, the basket mechanism was discontinued because donors were no longer willing to continue to channel funds through the Ministry of Health. Whilst the exact amount of international funding lost is unknown due to a move towards off-budget support, the reduction of development assistance for health from Sweden alone, from USD 8.1 million in 2009 to USD 680,000 in 2010, highlights the changes in the sector as a result of the event [11].

In examples like Zambia, criticism has been levelled at international donors for unduly impacting essential services, including primary care, by freezing funds, reverting to practices that do not reflect the aid effectiveness principles, forcing Government to change its approach to financing [10]. There has been significant discussion over whether the withdrawal of donor funding did impact health financing in the long term, and whether the reneging on aid coordination affected primary care financing. Sufficient time has passed since the 2009 corruption event in Zambia to start to understand how primary care financing changed over the period, in the context of the cessation of donor funding through the district basket mechanism.

By examining the financial allocations for the district level from 2006 to 2017, it is possible to determine how primary care financing changed over the period in which the corruption event occurred. This study aims to examine the budgets and actual expenditure allocated from central Government to the district level, for health, in Zambia from 2006 to 2017, to determine trends in funding for primary care. This analysis will allow us to examine if there were changes in government budgetary allocations and expenditure at district level, in the context of the withdrawal of donor funds.

A better understanding of the interaction between development assistance for health and government expenditure on health will contribute to our understanding of the level of fungibility of resources in the health sector in countries where development assistance for health constitutes a significant share of health spending. Fungibility describes to what extent government health expenditure is replaced by development assistance for health and vice versa.

## Methods

Primary care in Zambia encompasses all health services coordinated by 117 District Health Offices (DHOs), which include health services provided by health posts and centres, the community level, and district hospitals [12]. A ‘top down’ and ‘bottom up’ budgeting process occurs, whereby districts create costed annual work plans, and the central MoH provides a budget envelope for these plans [13].

The published budgets provide disaggregated data for each district. The district budgets are presented in a disaggregated form: personal emoluments or human resources (HR), health service delivery (HSD) and health systems management (HSM). The HR budget for each district is presented with the other district allocations (HSD and HSM), but is held and disbursed at the central level. The DHOs hold responsibility for managing and coordinating the rest of the budget: primary care HSD and HSM; known as the operational grant.

The budget for drugs is not presented by geography or level of care. Drugs are procured at the central level by the MoH, and pushed to districts, who do not have control over this funding. Instead, DHOs are allowed to use up to 4% of this operational grant to procure emergency drugs. We have decided not to include drugs in the analysis, because of the way the budget is presented.

Financial data were gathered from documents retrieved at the offices of the Zambian MoH and the Ministry of Finance (MoF). Budgetary allocations were taken from annual documents detailing estimates for each calendar year: ‘Yellow Book: Estimates of Revenue and Expenditure’ [14]; and actual expenditure, the resources spent in the financial year (January–December), were obtained from the annual documents entitled ‘Blue Book: Detailed Financial Report on Actual Expenditure’ [15].

According to the budget books provided by Government, HR includes wages, allowances and gratuities for individuals working at the primary care (district) level, and this is paid by the central MoH directly to health workers. The allocations are included in this analysis, because these resources are presented specifically for primary care, disaggregated by district. The operational grant (HSD and HSM) is disbursed from the central MoH to the DHOs. From here, the funds are either utilised by the DHO or disbursed to the facility level. Resources allocated to health service delivery are for first level referral, community health services, health centre clinical services, and health centre outreach. Resources allocated to Health System Management are for utilities, supervisory visits, administration, remuneration for contractual personnel and performance assessments. The sub-programmes included under HSD, HSM and HR, are presented each year in the yellow books.

The period of 2006 to 2017 has been chosen to allow for sufficient time to demonstrate the trends in primary care financing. Data collection took place between February and April 2016, in Lusaka, Zambia through manual data entry from books onto Microsoft Excel; and remotely in March 2019. Key informants and representatives from the MoH and the MoF assisted in identifying the necessary documents that contained the information required for this study.

Financial allocations were labeled according to their programme area: HR, HSD or HSM, the district, and year. HSD and HSM have been combined to provide the total operational grant that is disbursed to, and managed at, the district level. In 2013, the Zambian kwacha was rebased so that 1000 ZMK was the equivalent of 1 ZMW [16]. Therefore, figures prior to 2013 were converted into ZMW. The data have been adjusted for inflation using the consumer price index (CPI), with 2010 as the index year [17].

There were several changes to the way budgets were presented and allocated over the time period. The responsibility for primary care was moved from the MoH to the Ministry of Community Development, Mother and Child Health (MCDMCH) for 2013, but was then realigned back to the MoH in 2016 [18]. This changed where in the document the budgets were found, but not the budget lines. New districts have been created over the period of analysis: allocations for each district are included for the years in existence.

Microsoft Excel was used to analyse the data and identify trends over time for human resources and the operational grant. To establish the level of funding for primary care, the district allocations were summed to provide a national and regional picture. Financial data were also analysed per capita. Population figures and estimated growth rates were taken from the Population and Housing Census 2000 and 2010 and projected for each year between 2006 and 2017 [19].

The use of secondary data led to challenges regarding the quality of data. Civil servants in the MoH were hesitant as to whether actual expenditure data existed. Difficulties during the collection and location of data highlighted that detailed financial reports were not regularly compiled and used by the MoH for decision-making purposes, but were available and used by the MoF.

### Ethical considerations

No ethical approval was required for the study because it uses publicly available secondary data. Authority to conduct the study was obtained from the Permanent Secretary of the Ministry of Health, who provided support to the primary researcher to access the data in the form of the provision of documents and contacts.

## Results

**Figure 1.**
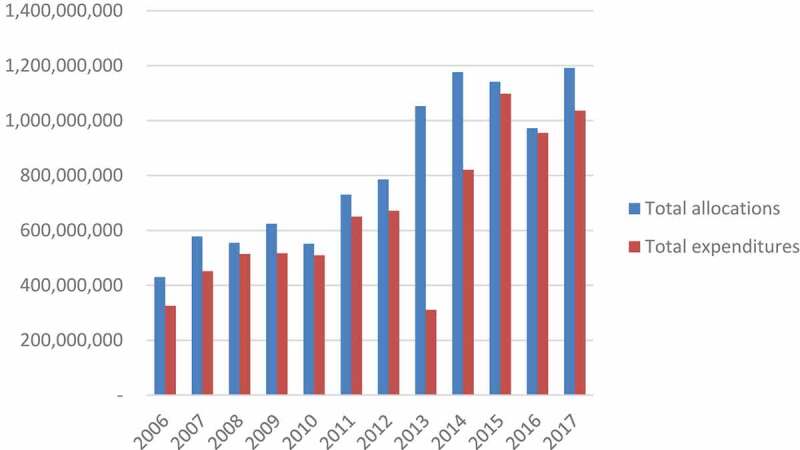
Total allocations and expenditures (including HR, HSD, HSM) for the district level, 2006–2017 (adjusted for CPI, ZMW)

Figure 1 demonstrates that the total budget and actual expenditure allocated to the district level increased from 2006 to 2017 by 177% and 165%, respectively. In 2008, 2010, and 2016 there was negative growth in the total budget for districts, declining by 4%, 12%, and 14% from the preceding years and in each case, recovering the following year. Actual expenditure decreased between 2009 and 2010; 2012 and 2013; and 2015 and 2016. The proportion of the total health budget allocated to districts has remained relatively consistent over the period of this analysis, at an average of 31%.

Actual expenditure sharply declined in 2013 by 54%. Numerous attempts have been made to understand this by consulting a variety of government and former government stakeholders. This data is consistent with what is reported in The World Bank Health Sector Public Expenditure Review, which attributed the large decline in expenditure to administrative reforms [20]. In 2013, responsibility for district level health care was transitioned from the MoH to the Ministry of Community Development, Maternal and Child Health.

**Figure 2.**
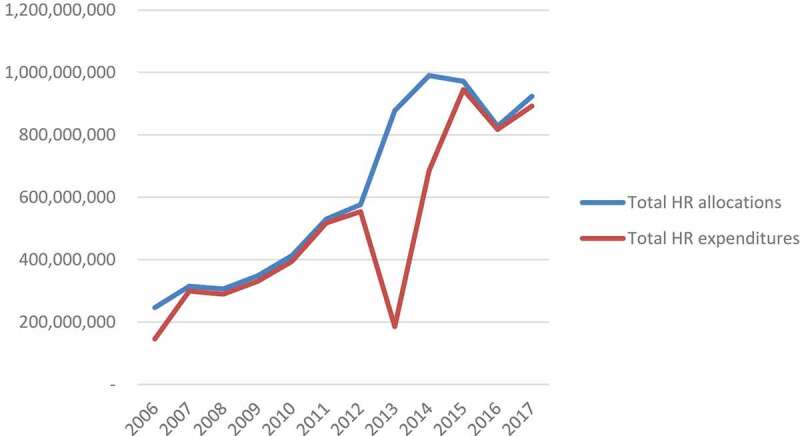
HR budget allocations and actual expenditure at the district level, 2006–2017 (Adjusted for CPI (2010 prices), ZMW)

Figure 2 demonstrates the increase in the HR budget and actual expenditure allocated to districts. The budget in 2017 was 3.7 times what it was in 2006: representing an average annual growth of 14%. The budget for HR experienced two declines: a reduction by 3% in 2008 and 15% in 2016. In 2006, HR accounted for 57% of the district budget and 45% of districts’ actual expenditure. By 2017, the proportion of resources spent on HR had increased to 78%. The HR budget has had a high execution rate from 2006 to 2017, with actual expenditure increasing six-fold over the period analysed. However, there is a reduced execution rate in 2013, demonstrated by the significant gap between budget and actual expenditure, in which only 21% of the budget was spent. This starts to recover in 2014 and is back up to 97% by 2015. The data demonstrates a substantial increase in the allocation of funds to human resources over the period.

**Figure 3.**
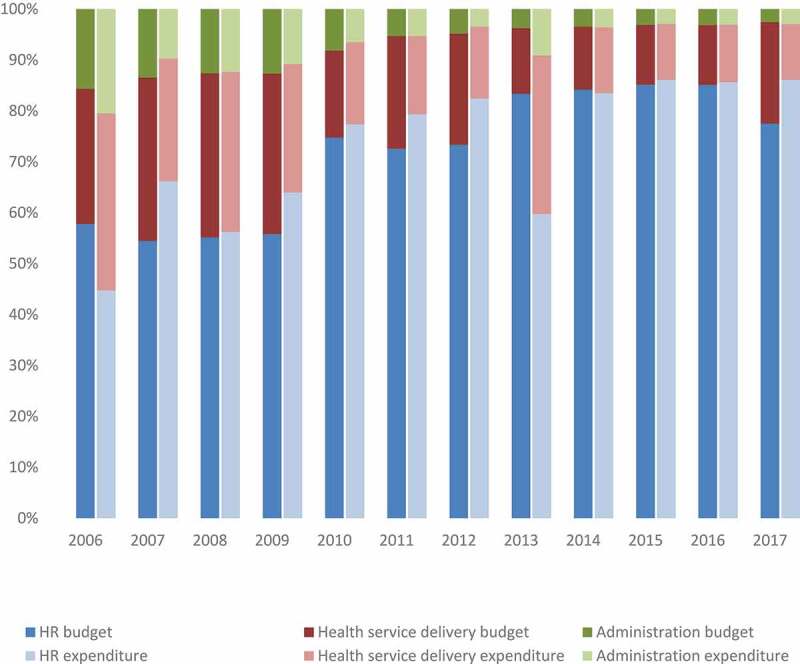
Proportion of primary care budget allocated and spent on HR, HSD, and HSM at the district level, 2006–2017 (Adjusted for CPI, ZMW)

The operational grant, which includes health service delivery and health systems management, is managed by the district and represents the resources that can be managed and spent at the decentralised level. Figure 3 highlights how the proportion of the overall budget for primary care allocated for the operational grant decreased over the period of analysis, from 42% in 2009 to 22% in 2017; despite the overall budget and expenditure for primary care increasing. The funding allocated and spent on the operational grant significantly reduced between 2006 and 2017. While the budget for the operational grant does increase in 2017 to its highest allocation in the period of analysis, this was not reflected in the actual expenditure, which decreased by 20% from 2006 to 2017.

The steep reduction in the budget between 2009 and 2010, and in actual expenditure between 2008 and 2010 in the operational grant coincides with the corruption event that occurred during this period. Following the corruption event and the subsequent cessation of donor funds, the operational grant expenditure does not recover to prior levels.

The decline in the operational grant allocated and spent by districts was experienced across districts, and regardless of population level. Table 1 presents the operational grant budget per capita, as an average for each region, demonstrating that the decline between 2009 and 2010 was experienced in all regions of Zambia. While the Northwestern region has consistently received the highest operational grant budget per capita, it too demonstrates the decline in 2010, followed by a swift recovery in 2011, and a gradual decline from 2011 onwards. The Copperbelt region, which had the lowest operational grant budget per capita from 2006 onwards, continued to have the lowest allocation in 2017 and also experienced the substantial decline in allocation between 2009 and 2010.

**Table 1.** Average operational grant per capita by region, budget and expenditure, 2006–2017 (Adjusted for CPI, ZMW)

However, the differences in the operational grant per capita between regions are less distinct when looking at expenditure. The range in operational grant per capita expenditure between the highest and lowest regions is only 4.8 kwacha in 2017, compared to a range of 22.2 kwacha for the range in the operational grant per capita budget.

## Discussion

Results from this study could be interpreted as an example of ‘reverse fungibility’, where withdrawal of development assistance for health was substituted with government spending. In the literature, fungibility has often been studied from the perspective of development assistance replacing government funding, and to what extent development assistance for health contributes to additional health spending. Limited attention, however, has been given to how governments mitigate disruption of development assistance for health.

The budget and actual expenditure allocated to districts in 2017 was over double that allocated in 2006: but, the rise in resources for HR more than tripled over the same period, accounting for this increase. The effects of the withdrawal of donor funding following the 2009 corruption event have been greatly debated[21,22]. These results demonstrate that the event did not affect overall resources for districts, but illustrate the changes to primary care over the period – made by both GRZ and donors.

Although overall resources for districts were unaffected, disaggregation of the data indicates that the withdrawal of funding did effect the operational grants provided for primary health care at the district level. Studies suggest that pooled funds, such as the district basket, are typically earmarked. In the case of Zambia, CPs could stipulate that their funds were used for the operational grant, and not HR. This explains why the withdrawal of funding from the district mechanism would have only affected the operational grant [23,24]. Where these conditions are stipulated, the operational grant, as opposed to HR, will be more sensitive to changes made to levels of funding [25].

Expenditure on human resources continued to increase despite the withdrawal of donor funds. On recognising the severe shortage of health workers in 2009, the Government prioritised HR, increasing the proportion of primary care funding allocated to HR over the period analysed [25]. Budget lines such as HR, infrastructure and drugs are difficult to default – for example, civil servants experience a high level of job security. Once resources are committed to HR, there is little freedom to renege on these commitments.

The decline in the operational grant indicates that districts’ ability to make financial decisions have diminished over the period. This is particularly evident from 2012 onwards, where data show that districts were allocated an average budget of ZMW14 per capita (adjusted for inflation), dependent on the district. This is the equivalent of US$ 1.14 per capita, which, according to the ‘yellow book’, must fund the district management team, district hospital, health facilities and community health posts within the area [26]. In reality, the operational grant per capita spent ranged from an average of US$0.52 in the Western and Copperbelt regions to US$0.83 in the Northwestern region – further reducing the ability of districts within these regions to fulfil planned spending on primary care.

It is unclear whether the Government of Zambia purposefully sought to fill the gap left by donors. The swift recovery in financing for primary care in 2011 suggests that the Government were proactive in seeking to fill the gap, but that this did not extend to ensuring that allocations for the operational grant were maintained. This highlights that while Government may have been responding to a reduction in primary care financing in general, they may not have been tracking what the impact of the shift of donor funding from on to off budget was having on specific programme areas, such as the operational grant, and thus, on district’s ability to make decisions close to the user.

Decentralisation reforms that were initially introduced in 1992, and subsequently strengthened through the National Decentralisation Policy in 2013, gave districts the autonomy to manage resources for primary care. This was intended to move decision-making closer to the end user, and, in doing so, enhance the quality of care [28]. While financial resources do not always result in increased decision-making authority, the Government’s inability to fill the gap left in the allocation for the operational grant that the districts are responsible for could diminish the intention of their decentralisation reforms. A retrospective study of the 1992 decentralisation reforms comments on the level of resource allocation for DHOs resulting in ‘moderate choices’: the same could be argued of the nominal operational grant provided to districts over the period of this study [27, p. v].

The World Bank Zambia Health Sector Public Expenditure Review showed that cooperating partner support continued to grow even after the 2009 corruption event [18]. In recent years, the majority of the CP funding has been directed to HIV/AIDS and other sexually transmitted diseases: in 2015 to 2016 these two areas accounted for up to 70% of donor funds [28]. CPs that withdrew from the district basket mechanism have continued to fund initiatives supporting primary care throughout this period, such as the DFID Tackling Maternal and Child Undernutrition 2 programme and the USAID funded Systems for Better Health programme in Zambia. However, these have been off budget [29,30]. The shift among donors from a pooled financing mechanism to a clearer project approach may result in negative implications for aid effectiveness. Aid effectiveness rests on five collectively agreed principles of good practice: ownership, alignment, harmonization, mutual accountability and results-based management [31]. However, the principles of ownership, alignment and harmonization are more difficult to uphold when working in a project approach. Previous studies have shown that government ownership over development interventions is restricted when funds are controlled by the donor [32].

Alignment and harmonization, included within the broader term of aid coordination, is complicated by stand-alone projects with a narrow focus, specific reporting requirements, and financial procedures [31]. The use of the off-budget modality during this period has affected Government ownership of the health sector: impacting the ability of Government to plan, coordinate, and implement its chosen priorities in the health sector [32]. This not only reduced aid effectiveness but reduced the efficiency of Government support, through increased duplication and a lack of harmonization amongst actors.

With the aim of encouraging CPs to reintroduce on-budget support; the Government of Zambia have introduced a Government Management Capacity Strategic plan [33]. This has been somewhat successful: in 2016, Sida reintroduced on budget support to the Zambian health sector through the Reproductive, Maternal, Child, and Adolescent Health and Nutrition Programme: channeling earmarked funding through the MoH [18,22]. Yet, while GRZ have been working to return to the arrangement prior to 2009, other donors in the health sector have experienced significant change in the aid policies of their own countries, with a move away from on-budget support and a desire to redefine aid effectiveness [34]. The GRZ may need to adapt to this changed context, focusing on ensuring that all CPs are ‘on plan’, even if they are not ‘on budget’.

### Limitations of the study

The results of this study rely on secondary data produced by the Ministry of Finance in Zambia. Discussions with stakeholders in the Ministry of Finance and the Ministry of Health highlighted that the expenditure data retrieved from the blue books were not used, or known about, by those outside of its production. Therefore, it raises questions regarding the extent to which this data is validated by the respective ministries.

A further limitation of this study was the constraints regarding triangulation. In order to strengthen the study, data should be collected from the district level to identify whether data reported at the central level correlate with data at the district level. This would also enable the study to identify patterns in the timing of disbursements, to further understand the bottlenecks in financing for primary care. This study could be complimented by additional studies exploring the decision-making of donors discontinuing support to the district basket, to determine if and how support to primary care in Zambia was continued. Future studies should also consider coupling the quantitative data with qualitative interviews at district and central level to identify the explanatory factors for the shift in donor modalities as well as the increased expenditure on behalf of the government of Zambia.

## Conclusions

This study aimed to examine the central budgetary allocations and expenditure at the district level for health in Zambia, to explore how primary care financing changed over the period of analysis, in the context of the 2009 corruption event. This paper demonstrates that while resources allocated and spent at the district level increased from 2006 to 2017, the human resources budget accounted for this increase and the operational grant declined.

This study highlights two important aspects. The first is that the government of Zambia was successful in quickly mitigating the financing gap for human resources for health that occurred as donors withdrew from the joint district funding mechanisms. The second learning is that the increase in government spending on human resources for health took place, at least partly, at the expense of district allocations in the operational grant. While the study did not look specifically into the explanations for this, it is reasonable to assume that human resources for health are inherently less flexible than operational grants, and therefore, that the operational grant, is more sensitive to decisions made by government or donors on funding allocations.

In a situation where on-budget support is no longer a possibility, both recipient and donor governments should look for new ways to implement the aid effectiveness principles, ensuring government ownership and alignment are continually prioritised; and that both are mindful of the effects of changes to funding on flexible budget lines in an effort to continue to provide primary health care and progress towards achieving health for all.

## Supplementary Material

Supplemental Material

## Data Availability

All data generated or analysed during this study are included in this published article [and its supplementary information files].

## Paper context

Volatile funding in the health sector can have implications for service delivery, in particular in low-income countries. Little is known about the consequences of sudden withdrawal of external funding from the health sector in a resources scarce setting. Our findings indicate that the government of Zambia managed to mitigate the withdrawal of external funding, by increasing overall allocations for district-level health care, but that this focused on human resources.

## References

[1] WHO. Declaration of Alma-Ata. International conference on primary health care; Alma-Ata, USSR: WHO; 1978.

[2] Starfield B, Shi L, Macinko J. Contribution of primary care to health systems and health. Milbank Q. 2005;83:457–8. PubMed PMID: 16202000; PubMed Central PMCID: PMCPMC2690145. 16202000 10.1111/j.1468-0009.2005.00409.x PMC2690145

[3] The Lancet. A renaissance in primary health care. Lancet. 2008;372:863. PubMed PMID: 18790289. 18790289 10.1016/S0140-6736(08)61369-0

[4] Cueto M. The promise of primary health care. Bull World Health Organ. 2005;83:322. PubMed PMID: 15976867; PubMed Central PMCID: PMCPMC2626224. 15976867 PMC2626224

[5] OECD. The Paris declaration on aid effectiveness. Paris: OECD; 2005.

[6] OECD. Aid effectiveness in the health sector: progress and lessons. Paris: OECD; 2012.

[7] Chansa C, Sundewall J, McIntyre D, et al. Exploring SWAp’s contribution to the efficient allocation and use of resources in the health sector in Zambia. Health Policy Plan. 2008;23:244–251. PubMed PMID: 18562459. 18562459 10.1093/heapol/czn013

[8] Lake S, Musumali C. 1999. Zambia: the role of aid management in sustaining visionary reform. Health Policy Plann. 2008;14:254–263. 10.1093/heapol/14.3.254 10621242

[9] Pereira J. Zambia: aid effectiveness in the health sector: a case study. Case study report, 2009. Brussels: Action for Global Health; 2009.

[10] Usher A. Donors lose faith in the Zambian health ministry. Lancet. 2010;376:403–404. 20700906 10.1016/s0140-6736(10)61205-6

[11] Open Aid. Sweden’s aid to Zambia for health sectors in 2016; 2017 [cited 2017 617]. Available from: https://openaid.se/aid/sweden/zambia/all-organisations/health/2016/

[12] Government of Zambia. Mid-term review of the implementation and performance of the revised national health strategic plan 2011–2016. Lusaka: Ministry of Health; 2015.

[13] Government of Zambia. Motions: report of the committee on estimates; 2014 [cited 2016 34]. Available from: http://www.parliament.gov.zm/node/557

[14] Government of Zambia. Estimates of revenue and expenditure for 2006–2016. Lusaka: Ministry of Finance.

[15] Government of Zambia. Detailed financial report on actual expenditure for 2006–2016. Lusaka: Ministry of Finance.

[16] Bank of Zambia. Currency rebasing technical guidelines. 2012. Lusaka: Bank of Zambia. (Contract No.: 2).

[17] World Bank. Consumer price index (2010=100); 2015 [cited 2016 53]. Available from: http://data.worldbank.org/indicator/FP.CPI.TOTL

[18] Embassy of Sweden. Health support for women, children and youth in Zambia: appraisal of intervention. Sida: Department for Organisational Development; 2015. (Contract No.: UF2014/11520).

[19] Government of Zambia. 2010 census of population and housing. Lusaka: National Statistical System; 2010.

[20] World Bank. Zambia health sector public expenditure review: 2006–2016. Washington (DC): World Bank; 2019.

[21] Usher AD. Key donors to reinstate health funding to Zambia. Lancet. 2015;386:519–520. PubMed PMID: 26293429. 26293429 10.1016/S0140-6736(15)61463-5

[22] Chansa C, Negin J. Key donors to reinstate health funding to Zambia. Lancet. 2015;386:2054–2055. PubMed PMID: 26700386. 10.1016/S0140-6736(15)00967-8 26700386

[23] Picazo O, Zhao F. Zambia health sector public expenditure review. Washington (DC): World Bank; 2009.

[24] Coppin E. Measuring good pooled funds in fragile states. London: Overseas Development Institute; 2012.

[25] Herbst CH, Vledder M, Campbell K, et al. The human resources for health crisis in Zambia. Washington (DC): World Bank; 2011.

[26] Oanda. Currency converter 2016. [cited 2016 101]. Available from: https://www.oanda.com/currency/converter/

[27] Bossert T, Chitah MB, Simonet M, et al. Decentralisation of the health system in Zambia. Partnerships for health reform. 2000. Maryland: Partnerships for Health Reform. (Contract No.: Major Applied Research 6 Technical Report 2).

[28] Ministry of Health. National health accounts 2013–2016. Lusaka: Ministry of Health; 2018.

[29] UK Department for International Development. DFID: DevTracker project GB-1-201927; 2017 [cited 2017 617]. Available from: https://devtracker.dfid.gov.uk/projects/GB-1-201927/transactions

[30] Abt Associates. Health systems strengthening interventions to improve Zambian health outcomes; 2015 [cited 2017 617]. Available from: http://www.abtassociates.com/Noteworthy/2015/Health-Systems-Strengthening-Interventions-to-Impr.aspx

[31] Leiderer S. Donor coordination for effective government policies? Helsinki: UNU WIDER; 2013.

[32] Sundewall J. Health sector aid coordination in Zambia. Stockholm: Karolinska Institutet; 2009.

[33] Government of Zambia. Governance management capacity strategic plan 2012–2016. Lusaka: Ministry of Health; 2012.

[34] Overseas Development Institute. Where next for development effectiveness? London: ODI; 2016.

